# Microwave-Assisted Synthesis of Pt/SnO_2_ for the Catalytic Reduction of 4-Nitrophenol to 4-Aminophenol

**DOI:** 10.3390/nano13172481

**Published:** 2023-09-02

**Authors:** Izabela Đurasović, Goran Štefanić, Goran Dražić, Robert Peter, Zoltán Klencsár, Marijan Marciuš, Tanja Jurkin, Mile Ivanda, Sándor Stichleutner, Marijan Gotić

**Affiliations:** 1 Laboratory for Molecular Physics and Synthesis of New Materials, Division of Materials Physics, Ruđer Bošković Institute, Bijenička c. 54, 10000 Zagreb, Croatia; idjuras@irb.hr (I.Đ.); goran.stefanic@irb.hr (G.Š.); ivanda@irb.hr (M.I.); 2 National Institute of Chemistry, Hajdrihova 19, SI-1001 Ljubljana, Slovenia; goran.drazic@ki.si; 3 Department of Physics, University of Rijeka, Radmile Matejčić 2, 51000 Rijeka, Croatia; rpeter@phy.uniri.hr; 4 Nuclear Analysis and Radiography Department, Centre for Energy Research, 1121 Budapest, Hungary; klencsar.zoltan@ek-cer.hu (Z.K.); stichleutner@izotop.hu (S.S.); 5 Division of Materials Chemistry, Ruđer Bošković Institute, Bijenička c. 54, 10000 Zagreb, Croatia; marijan.marcius@irb.hr; 6 Radiation Chemistry and Dosimetry Laboratory, Division of Materials Chemistry, Ruđer Bošković Institute, Bijenička c. 54, 10000 Zagreb, Croatia; tjurkin@irb.hr

**Keywords:** platinum, SnO_2_, microwave synthesis, catalyst, 4-nitrophenol, XPS, ^119^Sn Mössbauer

## Abstract

In this study, we present a new approach for the synthesis of Pt/SnO_2_ catalysts using microwave radiation. Pt(IV) and Sn(IV) inorganic precursors (H_2_PtCl_6_ and SnCl_4_) and ammonia were used, which allowed the controlled formation of platinum particles on the anisotropic SnO_2_ support. The synthesized Pt/SnO_2_ samples are mesoporous and exhibit a reversible physisorption isotherm of type IV. The XRD patterns confirmed the presence of platinum maxima in all Pt/SnO_2_ samples. The Williamson-Hall diagram showed SnO_2_ anisotropy with crystallite sizes of ~10 nm along the c-axis (< *00l* >) and ~5 nm along the a-axis (< *h00* >). SEM analysis revealed anisotropic, urchin-like SnO_2_ particles. XPS results indicated relatively low average oxidation states of platinum, close to Pt metal. ^119^Sn Mössbauer spectroscopy indicated electronic interactions between Pt and SnO_2_ particles. The synthesized samples were used for the catalytic reduction of 4-nitrophenol (4-NP) to 4-aminophenol (4-AP) in the presence of excess NaBH_4_. The catalytic activity of the Pt/SnO_2_ samples for the reduction of 4-NP to 4-AP was optimized by varying the synthesis parameters and Pt loading. The optimal platinum loading for the reduction of 4-NP to 4-AP on the anisotropic SnO_2_ support is 5 mol% with an apparent rate constant *k* = 0.59 × 10^–2^ s^–1^. The Pt/SnO_2_ sample showed exceptional reusability and retained an efficiency of 81.4% after ten cycles.

## 1. Introduction

Platinum nanoparticles (PtNPs) have garnered extensive attention due to their diverse applications across fields like biomedicine, energy conversion, storage, environmental cleanup, sensing, and catalysis [1,2,3,4,5,6,7,8,9,10,11,12]. Via the customization of synthesis techniques and conditions, a wide array of platinum nanoparticle shapes can be achieved, each possessing distinct attributes and potential uses [2]. Platinum can be harnessed in the form of nanoparticles dispersed within aqueous or organic mediums, or it can be distributed onto different substrates [2,3,4,5,6,7,8,9,10,11]. While PtNPs in a medium can aggregate, proving challenging to stabilize, dispersing PtNPs on substrates offers versatility for multiple applications. For instance, Papandrew et al. [3] crafted solid acid fuel cells using vapor depositing platinum from Pt(acac)_2_ onto solid acid CsH_2_PO_4_. Sized 2–4 nm, these PtNPs act as catalysts for oxygen reduction and electronic conductors in the electrode. In a separate study, Liu et al. [4] produced onion-like carbon nanospheres to anchor atomically dispersed platinum, serving as a catalyst for the hydrogen evolution reaction (HER). Calculations revealed a locally enhanced electric field at the curved support’s platinum site, boosting hydrogen evolution kinetics. Haneda et al. [5] explored Pt dispersion’s impact on oxidation reactions of carbon monoxide (CO) and propene (C_3_H_6_), prominent vehicle exhaust pollutants. Intriguingly, while CO oxidation turnover frequency (TOF) remained consistent across Pt/Al_2_O_3_ catalysts, C_3_H_6_ oxidation TOF surged with increased Pt dispersion. In a distinct context, Ponjavic et al. [6] showcased bacterial nanocellulose (BNC) as an effective eco-friendly support for high-performance PtNPs, particularly in methanol oxidation reactions. This sustainable catalytic system, leveraging BNC instead of conventional carbon-based materials, holds promise for broader implementation. Addressing hydrogen storage, Kostoglou et al. [7] synthesized plasma-derived nanoporous graphene, subsequently decorating it with PtNPs without altering surface chemistry or pore structure. These insights offer a pathway for innovative graphene-based nanocomposites suited for hydrogen storage applications under ambient conditions. Rioux and colleagues [8] delved into PtNPs incorporated within mesoporous SBA-15 silica, unearthing remarkable thermal stability for Pt/SBA-15 catalysts in pertinent turnover scenarios. Their approach outlines a blueprint for constructing various metal/support arrangements to decipher structure-selectivity connections in catalysis. Bai et al. [9] introduced a fresh avenue detailing the creation of ultrasmall PtNPs (averaging 0.9–2.3 nm) stabilized on hollow polymer nanoshells. This composite exhibited prowess as a catalyst for organic oxidation reactions of alcohols under ambient conditions. Size-dependent catalytic trends suggested peak activity in PtNPs of ≈ 1.7 nm, notably evident in the oxidation of 1-phenylethanol to acetophenone. However, PtNPs struggled to stabilize emulsions, leading to the emergence of irregular and sizeable polymer aggregates (>7 nm). Elezovic et al. [10] tackled oxygen reduction reactions, employing PtNPs on two distinct tin oxide-based supports, Sb-SnO_2_ and Ru-SnO_2_, in an acidic milieu. Both Pt/SnO_2_ catalyst types showcased akin catalytic prowess as Pt supported on commercially accessible carbon supports. Moreover, stability assessments unveiled minimal erosion of the electrochemically active surface area of Pt/SnO_2_ catalysts across repetitive cycles, highlighting their robustness for potential fuel cell applications. Smiechowicz et al. [11] investigated Pt/SnO_2_ traits and catalytic aptitude in CO oxidation, revealing the substantial influence of treatment conditions—temperature and atmospheric setting during catalyst heating—on active phase dispersion, surface layer composition, and O_2_ adsorption capacity of Pt/SnO_2_ catalysts. XRD analyses unveiled that H_2_-based heat treatment spurred the formation of Pt–Sn bimetallic compounds and assorted species, profoundly influencing CO oxidation catalyst activity. Martyla et al. [12] adopted a sol-gel technique to craft Pt/SnO_2_ systems, yielding high electrocatalytic activity post-low-temperature processing. The findings underscore the potential for facile generation of immensely active Pt/SnO_2_ electrocatalysts devoid of the need for thermal reduction conditions.

The choice of dispersion method depends on the particular requirements of the application and the nature of the medium into which the platinum nanoparticles are to be placed. Fine-tuning of the dispersion method is a critical factor in ensuring uniform distribution, stability, and desired properties of the dispersed nanoparticles. Ongoing research is therefore dedicated to the development of new synthesis techniques aimed at improving the catalytic properties of platinum and its effectiveness as a catalyst.

In this study, we use microwave radiation to decorate Pt/SnO_2_ with different Pt content by modulating the molar ratios of platinum and reducible SnO_2_ support. The platinum content was 1, 3, 5, 10, and 15 mol%, corresponding to a ratio [Pt^IV^/(Pt^IV^ + Sn^IV^)] of up to 0.15. Our studies address platinum dispersions, physicochemical properties, and catalytic and reusable properties of Pt/SnO_2_ in the context of the reduction of 4-NP to 4-AP.

Our study presents a new approach for the synthesis of Pt/SnO_2_ catalysts using microwave radiation, which allows the controlled formation of platinum particles on an anisotropic SnO_2_ support. The resulting samples possess mesoporous properties with uniformly dispersed PtNPs in the micrometer range. These catalysts exhibit exceptional efficiency in the catalytic reduction of 4-nitrophenol to 4-aminophenol, even after 10 cycles, with optimal performance achieved at a Pt loading of 5 mol%. The results suggest promising applications in catalysis, adsorption, and gas sensing, with the potential for advanced catalytic systems in various environmental and catalytic scenarios.

## 2. Materials and Methods

### 2.1. Chemicals

Tin(IV) tetrachloride (Product No. 244678), hexachloroplatinic acid (Cat. No. 152509), and ammonium hydroxide (Product No. 221228) produced by Sigma Aldrich were used as received. Chemicals used for catalytic experiments were: 4-nitrophenol—Sigma Aldrich, Reagent Plus, ≥99%, CAS: 100-02-7, Product No.: 241,326 and sodium borohydride (NaBH_4_)—Alfa Aesar, min.98%, CAS: 16940-66-2, Product No.: 88983. Deionized Milli-Q water was used in catalytic experiments.

### 2.2. Stock Solution Preparation

The SnCl_4_ solution was prepared by weighing 35.06 g of the solid SnCl_4_·5 H_2_O and mixing it with 50 mL of deionized Milli-Q water. The H_2_PtCl_6_ solution was prepared by mixing 1 g of the solid H_2_PtCl_6_ with 986 µL of deionized Milli-Q water. The calculated concentrations of both the tin stock solution and the platinum stock solution were 2.0 mol dm^–3^ (2M SnCl_4_ and 2M H_2_PtCl_6_).

### 2.3. Synthesis of the Samples

From previously prepared stock solutions, appropriate amounts of the aliquots were taken so that the molar ratio of tin and platinum ions in the mixture was from 0.00 to 0.15 (Table 1). For example, for the synthesis of the SnO_2_ sample doped with 15 mol% of Pt^IV^, i.e., at a ratio [Pt^IV^/(Pt^IV^ + Sn^IV^)] = 0.15, the 450 µL of H_2_PtCl_6_ stock solution was added to 2.55 mL of SnCl_4_ stock solution. The aliquots were added to a 100 mL glass and diluted with 17 mL of deionized Milli-Q water. The solution was stirred with a magnetic stirrer for 15 min before 1 mL of 1 M ammonia was added to a stream and stirred for an additional 15 min. The pH of the solutions was measured using a pH meter before and after the addition of ammonia, and the initial pH of the solutions increased from 0.5–0.7 up to 0.9–1.1. The magnet was removed from the glass, and the newly formed suspension was quantitatively transferred to a glass cuvette, which was inserted in a microwave digestion platform. Microwave-assisted hydrothermal synthesis took place for 30 min at 230 °C. The suspension was then quantitatively transferred to a Petri dish and placed overnight in an oven at 90 °C in order to evaporate the residual water. The precipitate was scraped from the Petri dish and homogenized in a mortar and pestle. It was then heated in air at 400 °C for 2 h in a tube furnace before being used for analysis and characterization.

### 2.4. Instrumental Analysis

X-ray diffraction (XRD) measurements were performed at room temperature using an APD 2000 diffractometer (CuKα radiation, graphite monochromator, NaI-Tl detector) manufactured by ITALSTRUCTURES, Riva Del Garda, Italy.

Scanning electron microscopy was performed on Jeol Ltd. (Tokyo, Japan). 700F field-emission scanning electron microscope coupled with EDS/INCA 350 system for energy dispersive x-ray spectrometry manufactured by Oxford Instruments Ltd. (Abingdon, UK).

This study utilized an Atomic Resolution Scanning Transmission Electron Microscope (AR STEM), specifically the Jeol ARM 200 CF model, operating at 200 kV. This instrument was coupled with the Gatan Quantum ER system, incorporating capabilities for electron energy loss spectroscopy and energy dispersive X-ray spectrometry using the Jeol Centurio 100 module.

For the nitrogen adsorption analysis conducted at 77 K, the Quantachrome Autosorb iQ3 system was employed, employing the Brunauer-Emmett-Teller (BET) technique to assess the material properties. Prior to testing, a controlled heating process up to 250 °C was administered under vacuum conditions to eliminate residual gases and moisture. The evacuation process continued until the pressure variation ceased to rise rapidly, achieving a level below 50 millitorr per minute. Subsequent adsorption and desorption isotherm measurements at 77 K were performed across a relative pressure span encompassing approximately 10^–5^ up to nearly 0.99.

To examine the oxidation state of Sn and Pt in the Pt/SnO_2_ samples, X-ray photoelectron spectroscopy (XPS) was employed. The analysis was conducted under ultra-high vacuum (UHV) conditions, utilizing a SPECS instrument. The experimental setup utilized an excitation energy of 1486.74 eV derived from Al Kα X-ray emission and the Phoibos100 electron energy analyzer. In order to neutralize charge accumulation in nonconductive oxide samples, a 5-eV electron flooding method was applied during the XPS analysis. During the evaluation of Pt 4f core levels, a pass energy of 50 eV was selected, whereas for Sn 3d level spectra, a pass energy of 10 eV was employed. The fitting of experimental data curves was carried out using a combination of Gaussian and Lorentzian functions via Unifit software [13]. All photoemission spectra were calibrated utilizing the C 1s peak located at a binding energy (BE) of 284.5 eV.

^119^Sn Mössbauer spectra were measured at room temperature in transmission geometry by using a standard WissEl Mössbauer spectrometer setup along with a ^119m^Sn(CaSnO_3_) radioactive source (RITVERC JSC) with an activity of ~2.2 mCi. The source movement followed a sinusoidal velocity signal with a velocity extrema of ±6.13 mm s^–1^. The unfolded spectra were recorded into 2048 channels that were subsequently folded into 1024 channels for analysis and further processing. Isomer shift (*δ*) values are quoted with respect to a SnO_2_ reference powder (Merck) whose isomer shift can be taken to be equal to that of the CaSnO_3_ source matrix. The velocity axis was calibrated by measuring the reference SnO_2_ (*δ* = 0 mm s^–1^) powder together with β-Sn (*δ* = 2.56 mm s^–1^). For the Mössbauer measurements, circular absorbers with a diameter of 15.5 mm were prepared by mixing 15 mg of the original powders evenly with ~100 mg of cellulose used as filler material. The recorded spectra were analyzed with version 4.0i of the MossWinn program [14] by assuming the validity of the thin absorber approximation.

UV–vis reflectance spectra were collected with a Shimadzu UV/VIS/NIR spectrometer, model UV-3600. The used wavelength range was from 600 to 200 nm.

### 2.5. Catalytic Measurements

We examined the catalytic reduction of 4-nitrophenol (4-NP) to 4-aminophenol (4-AP) using UV-visible spectrophotometry with the presence of our synthesized samples and NaBH_4_. Before catalytic measurements, the solutions containing 4-NP and NaBH_4_ were not purged with nitrogen gas. The NaBH_4_ solution in water was freshly prepared prior to each experiment. Typically, we mixed 0.3 μmol of 4-NP (20 μL from a 0.015 M solution) with 2.7 mL of ultrapure water in a quartz cuvette. Then, we added 79.3 μmol of NaBH_4_ (20 μL from a 0.793 M solution). To this solution, we introduced 20 μL of the catalyst (at a concentration of 3 mg/mL in ultrapure water) using a micropipette, and we mixed it quickly. Right after adding the synthesized samples, we measured the UV-visible absorption spectra at specific time intervals until the nitrophenolate ions’ maximum absorption at 400 nm disappeared. The transformation into 4-AP was tracked by observing an increase in the maximum absorption at 300 nm.

Recyclability (reusability) tests were performed using the settings described above. After each cycle, 0.3 μmol of 4-NP (20 μL of 0.015 M solution) was added to the suspension and rapidly mixed, after which UV-vis spectra were collected in the same manner. This was performed for a total of 10 cycles. Before the fourth and seventh cycles, 79.3 μmol of NaBH_4_ (20 μL of 0.793 M solution) was also added to the 4-NP solution to ensure that NaBH_4_ was in large excess.

## 3. Results

### 3.1. XRD Results

**Figure 1:**
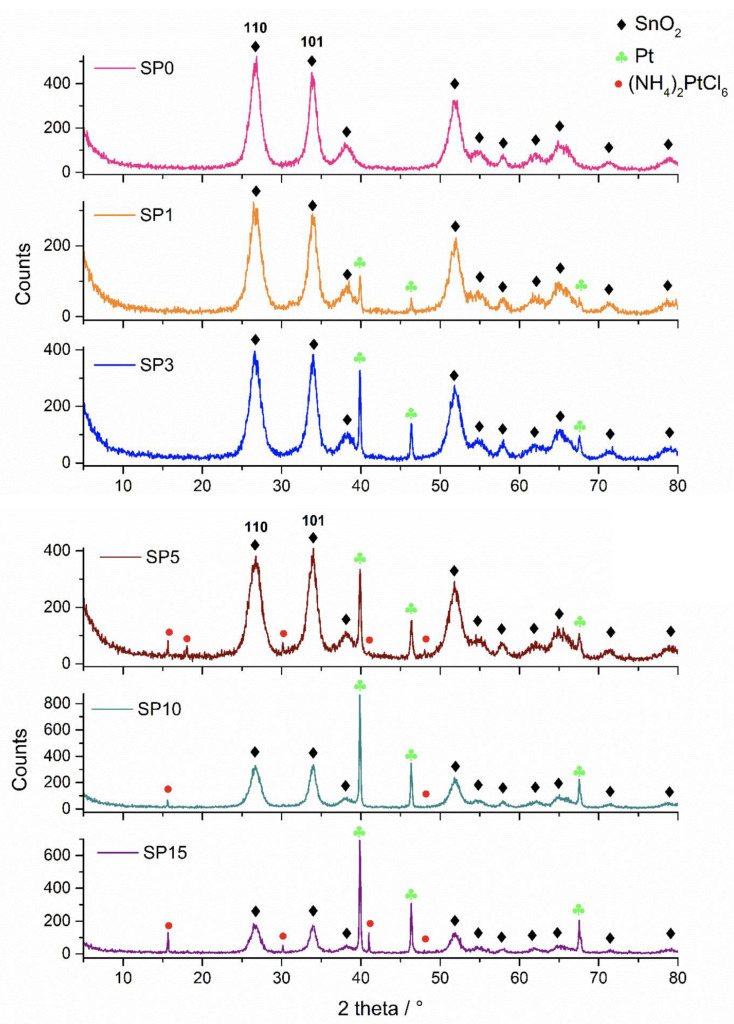
XRD patterns of samples SP0, SP1, SP3, SP5, SP10, and SP15.

Figure 1 shows the XRD patterns of the synthesized samples. In the sample without platinum (SP0), only cassiterite was found in the XRD patterns. In addition to cassiterite, platinum was found in samples SP1, SP3, SP5, SP10, and SP15, while (NH_4_)_2_[PtCl_6_] was found in samples SP5, SP10, and SP15. The results of the phase analysis are summarized in Table 2.

**Figure 2:**
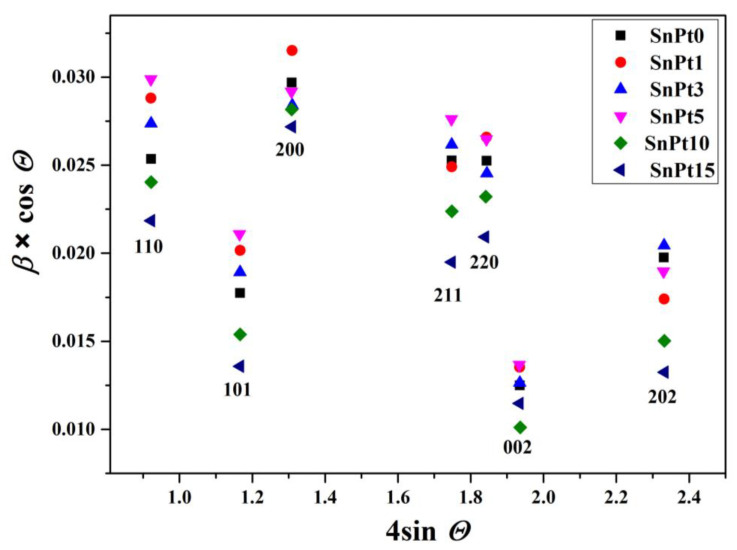
Williamson-Hall plot of the most prominent well-separated diffraction lines of the SnO_2_ phase in samples SP0, SP1, SP3, SP5, SP10, and SP15.

The Williamson-Hall plot [15] indicated the presence of size anisotropy with significantly narrower diffraction lines along the direction <*00l*> compared to the direction <*h00*> (Table 3 and Figure 2). Due to the pronounced size anisotropy, the values of crystallite sizes in several different *hkl* directions were estimated from the Scherrer equation:

(1) $$D_{\mathrm{hkl}}=\frac{0.9\lambda }{\beta \mathrm{hkl}\;\times \cos\theta }$$

where *D_hkl_* is a volume average of the crystal thickness in the direction normal to the reflecting plane *hkl*, *λ* is the X-ray wavelength (CuKα), *θ* is the Bragg angle, and *β_hkl_* is the pure full width of the diffraction line (*hkl*) at half the maximum intensity. The values of *β_hkl_* were found from the observed full width at half the maximum intensity (*B_hkl_*) of the diffraction lines after correction for instrumental broadening for which the corresponding width of the diffraction lines of well crystalline ZnO sample was used [16]. The *B_hkl_* values of the diffraction lines were determined using the individual profile fitting method (computer program XFIT [17]).

### 3.2. FE SEM Results

**Figure 3:**
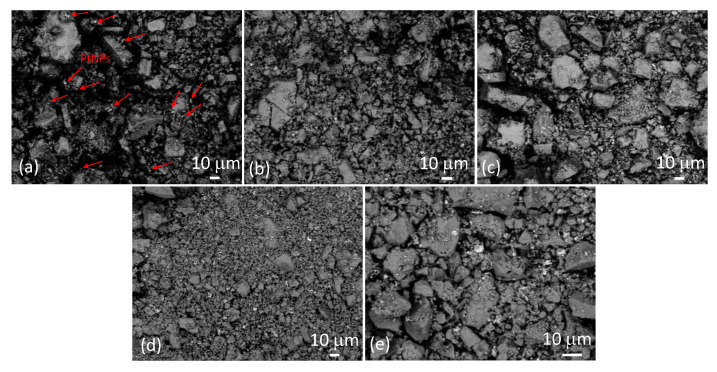
SEM images of samples SP1 (**a**), SP3 (**b**), SP5 (**c**), SP10 (**d**), and SP15 (**e**) at low magnification were taken with a backscatter detector. Some of the bright spots in sample SP1 (**a**) corresponding to PtNPs are marked with arrows as an example, while the bright spots in the other samples are not marked.

Figure 3 shows SEM images of samples SP1 to SP15 at low magnification, taken in backscatter mode. The much brighter irregular spots correspond to platinum. In Figure 3a, these spots are represented using arrows due to the relatively low platinum content in sample SP1 (1 mol%), while they are clearly visible in the other samples. It can be seen that these relatively large PtNPs are dispersed throughout the sample. The backscattered electron (BSE) signal intensity is approximately proportional to the atomic number (Z) of the elements, with an exponent of around 1.7. Consequently, heavier elements like platinum (Z = 78) exhibit significantly brighter BSE images compared to lighter elements like tin (Z = 50). This relationship contributes to the contrasting brightness observed in SEM-backscattered images of different elements.

**Figure 4:**
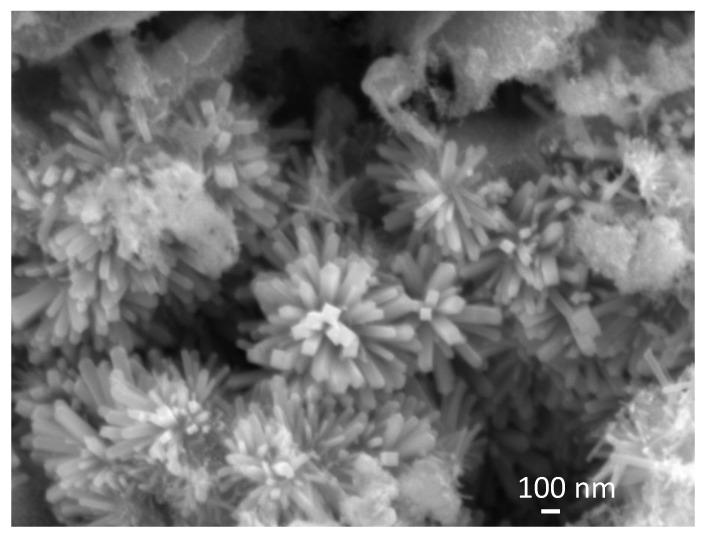
SEM image of sample SP5. A detail shows urchin-like anisotropic particles.

Figure 4 shows an SEM image of sample SP5 that, along with big irregular particles (see Figures S4 and S7 in the Supplementary Materials), contains urchin-like anisotropic particles.

**Figure 5:**
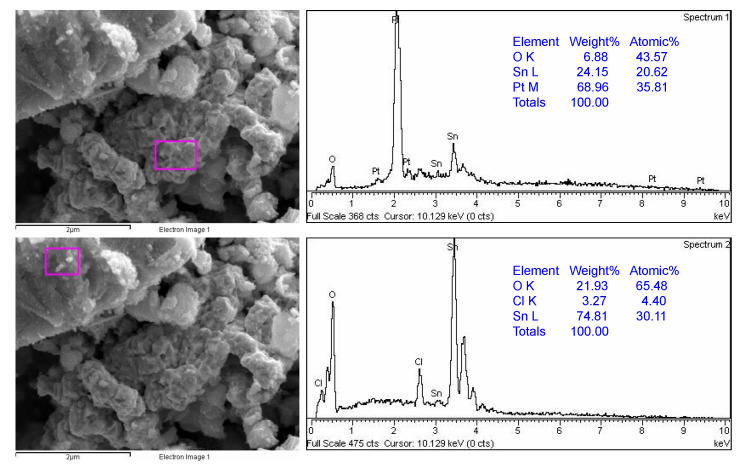
SEM-EDS results of sample SP1. EDS analyses were taken from two different marked sites showing the platinum-rich region (**upper panel**) and SnO_2_-rich region (**lower panel**).

Figure 5 shows the SEM-EDS results of sample SP1. Two marked sites can be seen, one rich in platinum (Pt) and the other rich in tin (Sn). The EDS results for samples SP3, SP5, SP10, and SP15 can be found in the Supplementary Materials. The results show that the platinum in the Pt/SnO_2_ samples is heterogeneously dispersed in the nanometer range.

### 3.3. STEM Results

**Figure 6:**
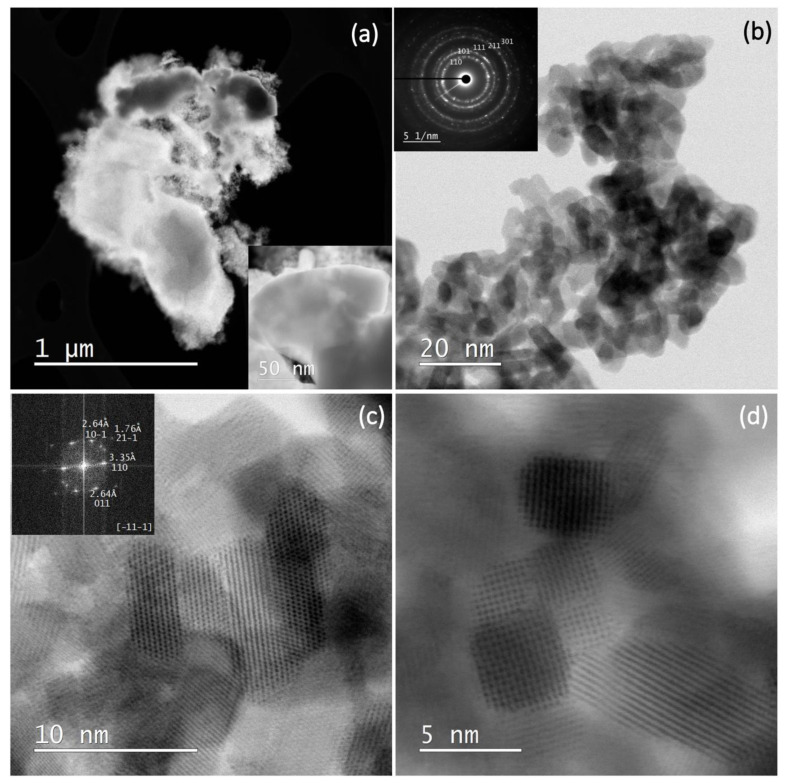
STEM DF image at low magnification (**a**); STEM BF image at higher magnification with a SAED image in inset, the powder patterns are indexed to SnO_2_ (cassiterite) (**b**); a high-resolution image of SnO_2_ with an FFT image in the (-11-1) zone (inset) (**c**); a high-resolution image of several SnO_2_-NPs with clearly visible lattice fringes (**d**).

Figure 6 shows the STEM and SAED (selected area electron diffraction) results of sample SP3. Figure 6a shows the STEM dark field (DF) image at low magnification. Bright spots corresponding to heavy elements such as platinum can be seen in the DF image. However, in the present case, these platinum-rich areas appear as dark areas due to the large and thick platinum particles in the upper part of the image (see also inset), while the small SnO_2_ nanoparticles appear as bright areas. Figure 6b shows a bright-field image (BF) at higher magnification with a SAED image in the inset. The powder patterns are indexed to SnO_2_ (cassiterite). Figure 6c shows a high-resolution image of SnO_2_ with an FFT (Fast Fourier Transform) image in the (-11-1) zone (inset). The patterns appear as bright dots in the FFT image, and the indexed pattern 011 with 2.64 angstroms, for example, corresponds to the (011) crystallographic planes of SnO_2_ with a lattice spacing of 2.64 angstroms. Figure 6d shows a high-resolution image of several SnO_2_-NPs with clearly visible lattice fringes.

**Figure 7:**
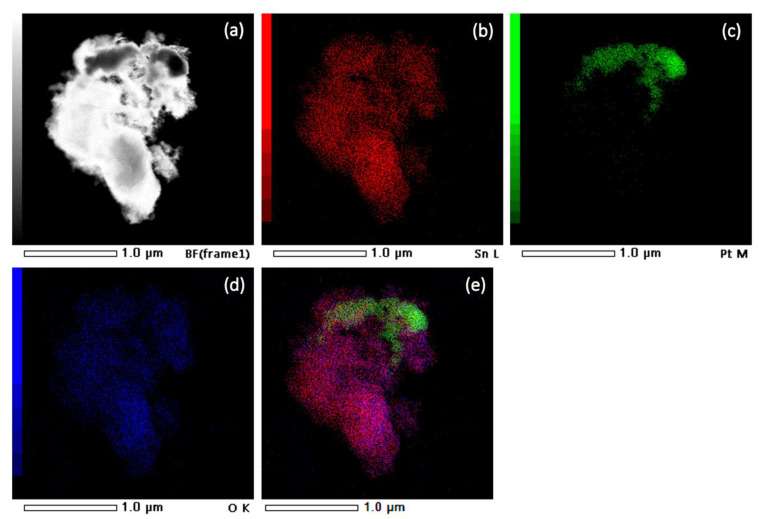
STEM image of sample SP3 (**a**) and corresponding EDXS elemental mapping images of Sn L edge (**b**), Pt M edge (**c**), O K edge (**d**), and overlay of Sn L, Pt M, and O K edges (**e**).

Figure 7 shows a STEM image and EDXS analysis of sample SP3. The corresponding EDXS elemental mapping images of the Sn L edge (b), the Pt M edge (c), the O K edge (d), and the superposition of the Sn L, Pt M, and O K edges (e) can also be seen and show significant clumping of Pt, indicating a non-uniform dispersion of all elements in the samples.

### 3.4. BET Nitrogen Adsorption–Desorption Isotherm and Pore Volume

**Figure 8:**
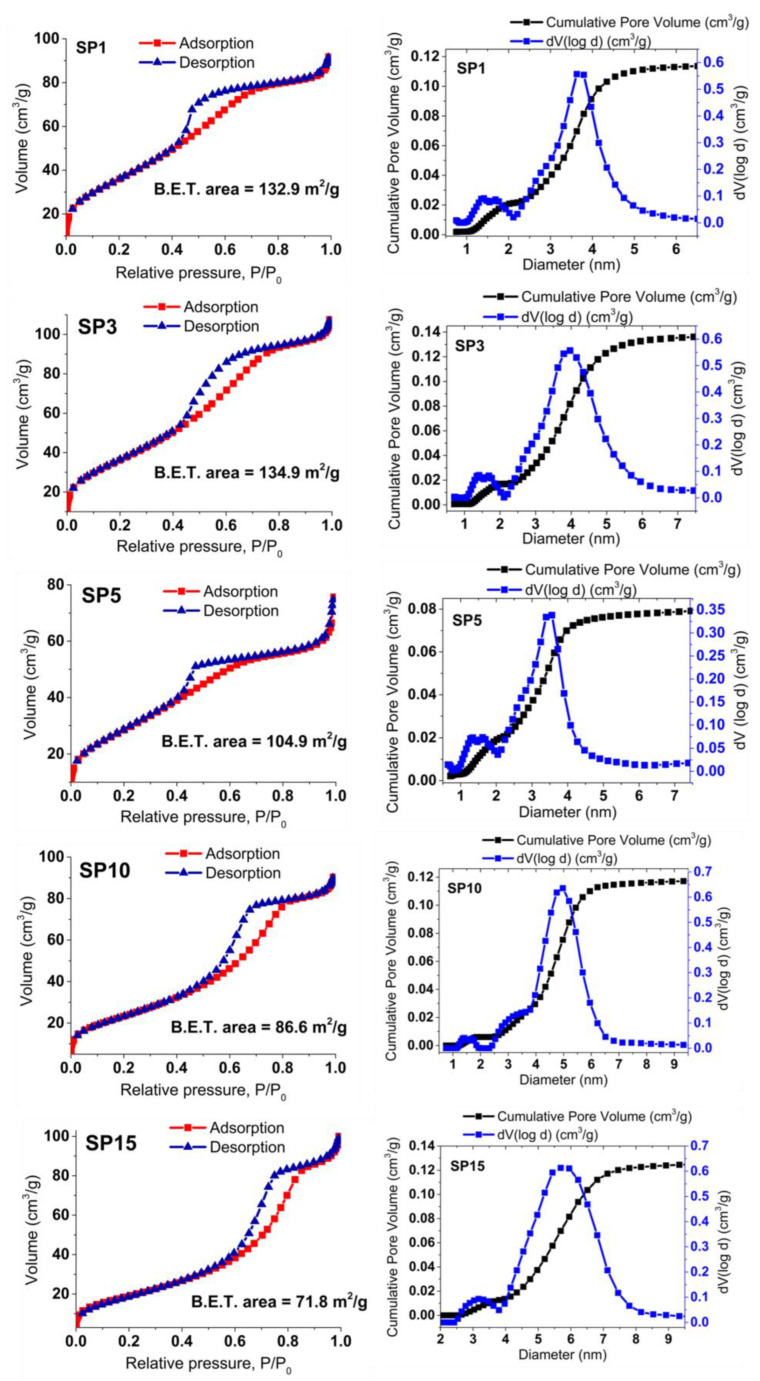
Displays nitrogen (N_2_) gas adsorption (red line, squares) and desorption (blue line, triangles) isotherms for the synthesized samples, along with calculated BET surface areas. The accompanying pore volume distribution offers insights into material porosity.

In Figure 8, the gas (N_2_) adsorption (depicted using the red line and squares) and desorption (illustrated with the blue line and triangles) isotherms for the SP0 to SP15 samples are showcased, alongside the corresponding analysis of pore volume distribution. These samples exhibit reversible physisorption isotherms classified as type IV, indicating their mesoporous nature. The evaluation of Brunauer, Emmett, and Teller (BET) specific surface areas reveals a declining trend in the BET surface area across the samples, ranging from 134.1 m^2^/g for the SP3 sample to 71.8 m^2^/g for the SP15 sample. The analysis of pore volume distribution across all samples consistently supports their mesoporous characteristics, with an average pore diameter of approximately 4 nm.

### 3.5. XPS Results

**Figure 9:**
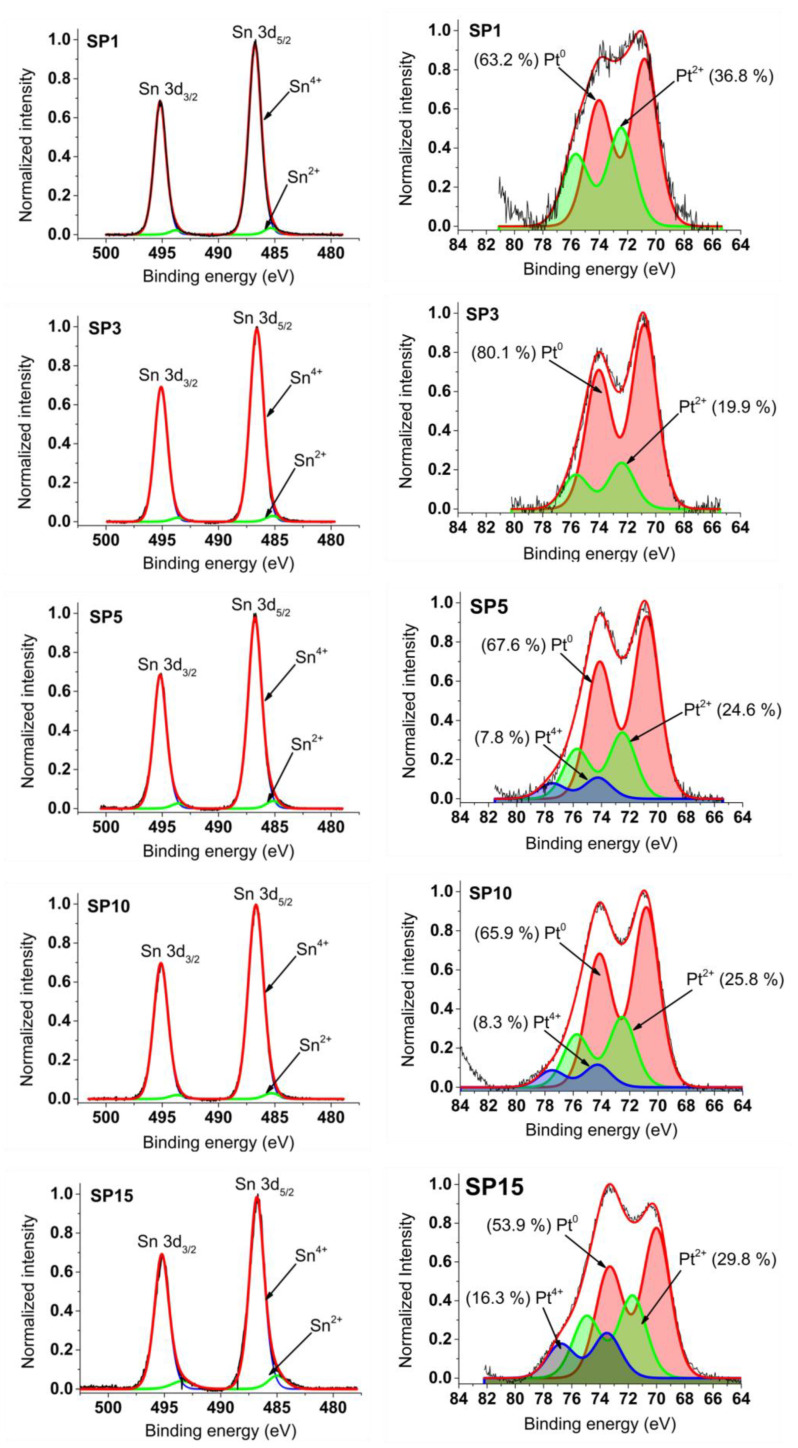
XPS spectra measured around Sn 3d and Pt 4f core levels of synthesized samples.

In the left part of Figure 9, the graphs depict photoemission spectra encompassing the Sn 3d core levels within the platinum-loaded SnO_2_ samples. These spectra exhibit a dual spin-orbital doublet pattern, corresponding to distinct oxidation states of tin—Sn^2+^ and Sn^4+^. The dominant signal stems from Sn atoms in the Sn^4+^ state (SnO_2_), evidenced using the Sn 3d_5/2_ peak positioned around 486.7 eV binding energy (BE) and the 3d_3/2_ peak shifted by 8.4 eV towards higher BE.

Moving to the right panel in Figure 9, Pt 4f photoemission curves of the SnO_2_ samples with platinum loading are displayed. These spectra are meticulously fitted with two or three doublets, ascribed to platinum oxidation states: Pt^0^, Pt^2+^, and Pt^4+^. The energy positions of Pt 4f_7/2_ peaks emerge at approximately 70.8 eV (Pt^0^), 72.5 eV (Pt^2+^), and 74.3 eV (Pt^4+^). Notably, the energy separation between Pt 4f_7/2_ and Pt 4f_5/2_ peaks is consistent at around 3.2 eV across all Pt oxidation states, aligning well with existing data on platinum oxides from previous literature.

The refined Pt 4f and Sn 3d spectra guide the determination of peak positions and relative contributions (%) of Pt^4+^, Pt^2+^, Pt^0^, Sn^4+^, and Sn^2+^ within the synthesized samples. Comprehensive details of these outcomes, including numerical values, are provided in Supplementary Table S1. Meanwhile, Table 4 encapsulates the XPS-derived average oxidation states (AOS) of platinum (Pt) and tin (Sn) achieved in this investigation, juxtaposed with findings from reference [18]—a comparison elaborated upon in the ensuing discussion.

### 3.6. Mössbauer Spectroscopy

**Figure 10:**
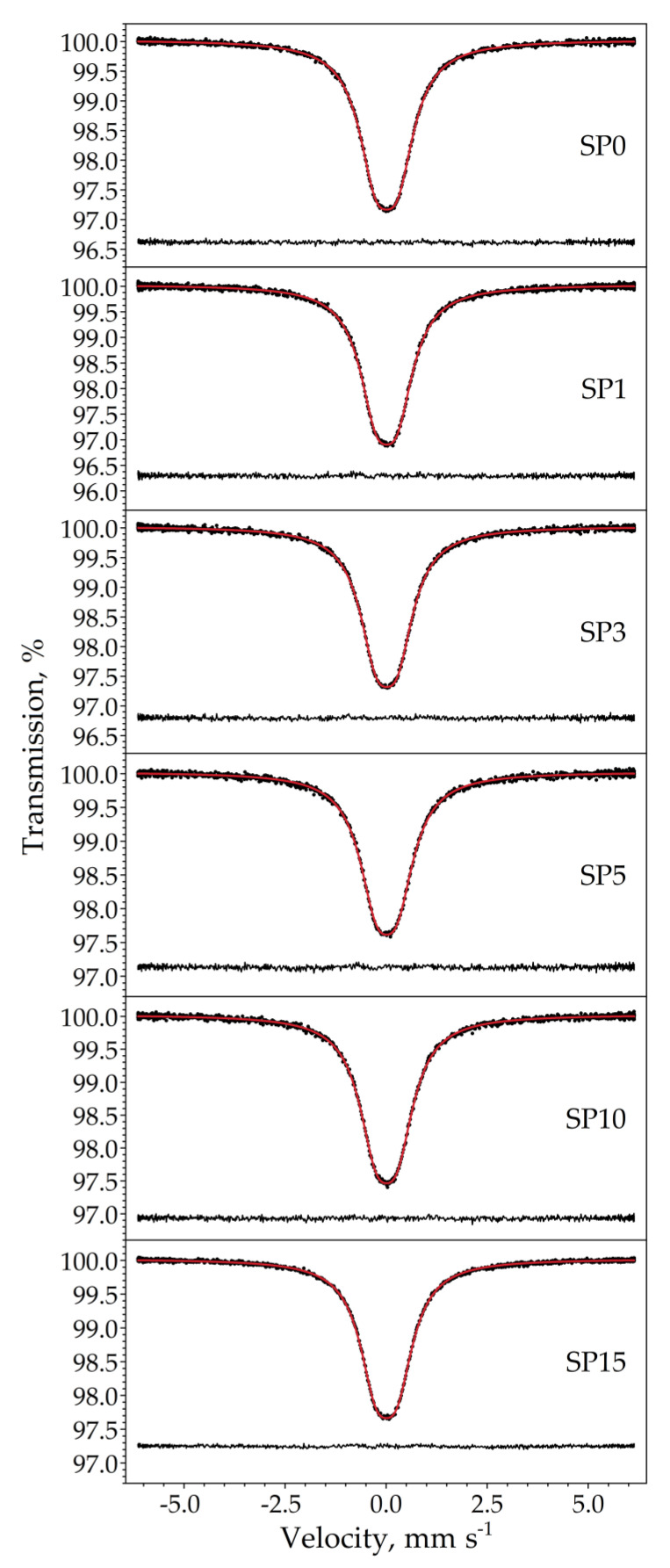
Room temperature ^119^Sn Mössbauer spectra (dots) of the samples SP0–SP15 along with the envelope (solid line) of the Lorentzian quadrupole doublet fitted to it. The fit residual is drawn below the spectra.

**Figure 11:**
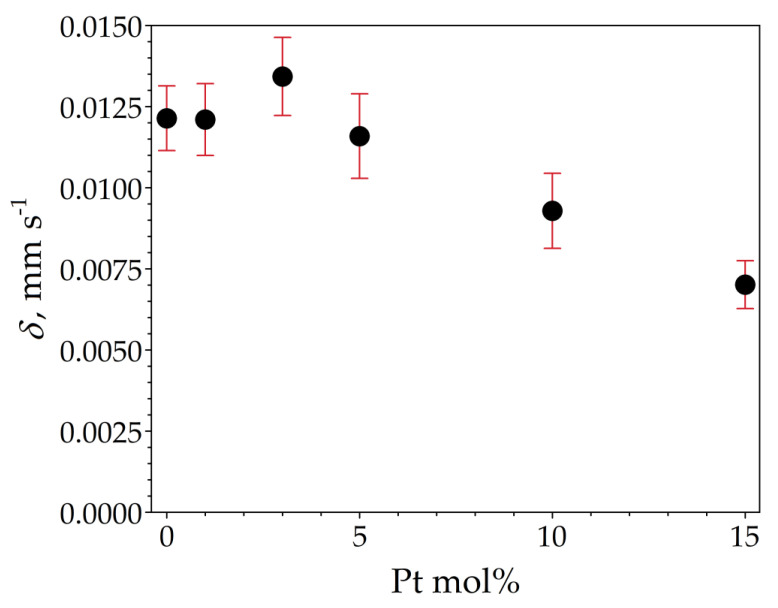
^119^Sn isomer shift (*δ*) depicted as a function of Pt molar concentration of the samples SP0 to SP15, with vertical error bars indicating the standard statistical error (±1σ).

The room temperature ^119^Sn Mössbauer spectra of the samples are displayed in Figure 10. The spectra were well fitted with a single symmetrical quadrupole doublet of Lorentzians, with the normalized chi-square of the fit being close to 1 in all the cases. The obtained Mössbauer parameters are listed in Table 5. The near-zero isomer shift values confirm that tin is present in the oxidation state of Sn^4+^. Absorption signals from stannous compounds were not detected. The obtained quadrupole splitting and line width values correspond well to those expected for crystalline SnO_2_ (see, e.g., [19]) and thereby confirm the formation of cassiterite. There are small differences in the isomer shift values among the samples (Table 5), which nevertheless reveal a decreasing tendency with increasing Pt concentrations, as depicted in Figure 11.

### 3.7. Catalytic Measurements

**Figure 12:**
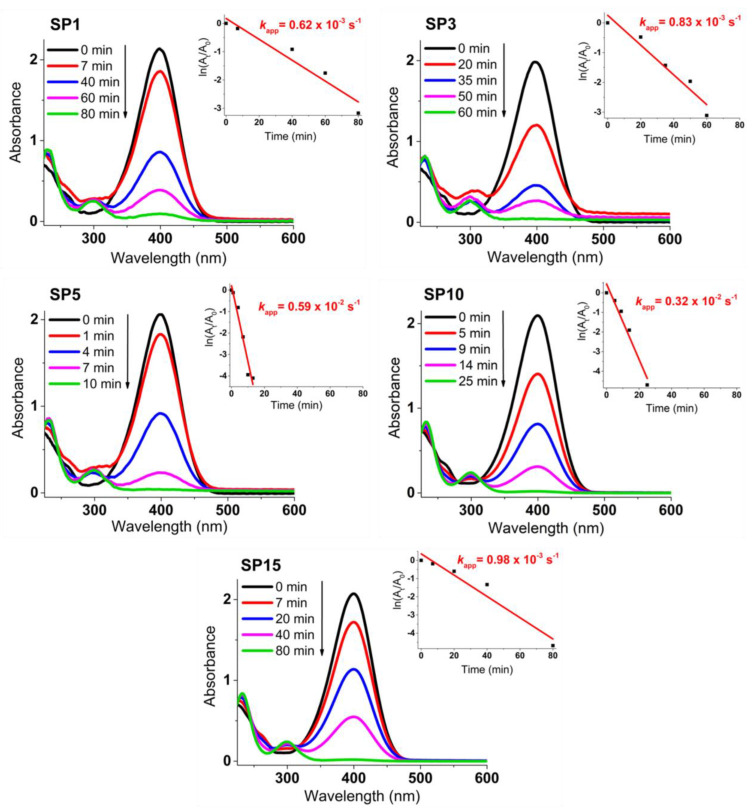
Time-dependent catalytic reduction process of 4-nitrophenol (4-NP) to 4-aminophenol (4-AP) using platinum-decorated SnO_2_ (SP1 to SP15) samples. The insets present the ln(A*_t_*/A_0_) plot against reaction time, showcasing the calculated rate constant values (*k*_app_ in s^–1^) derived from the slopes of the linear segments.

Figure 12 presents the progress of catalytic reduction from 4-nitrophenol (4-NP) to 4-aminophenol (4-AP) with excess NaBH_4_ over time, utilizing platinum supported on SnO_2_ (SP1 to SP15 samples). The insets showcase the ln(A*_t_*/A_0_) plot against reaction time. The depicted values of the rate constant *k*_app_ (s^–1^) stem from the linear segment slopes, founded on a pseudo-first-order kinetic equation:

ln(*C_t_*/*C*_0_) = ln(A*_t_*/A_0_) = − *k*_app_ × t (2)

The untreated SP0 sample, void of added platinum, is entirely inactive for the catalytic reduction of 4-NP to 4-AP (refer to Supplementary Materials). Conversely, samples carrying loaded platinum display robust catalytic effectiveness. Among them, the 5-mol% platinum-supported SnO_2_ (sample SP5) emerges as the most catalytically potent for 4-NP to 4-AP reduction, marked by a *k*_app_ rate constant of 0.59 × 10^–2^ s^–1^. This catalytic proficiency mirrors the decline in absorbance at 400 nm due to 4-nitrophenolate ions and the concurrent rise in absorbance at 300 nm due to 4-aminophenol formation (see Supplementary Materials).

**Figure 13:**
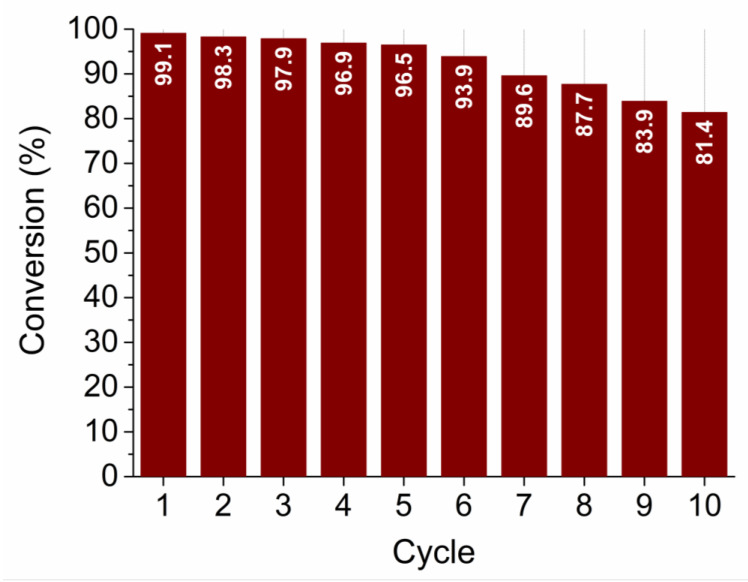
Recyclability (reusability) test of sample SP5 for 10 cycles.

The recyclability (reusability) test was performed for the sample SP5 with the highest catalytic activity. The test was performed in the same way as the other catalytic measurements for a total of 10 cycles. As can be seen in Figure 13, sample SP5 is very robust and exhibits very high efficiency in reducing 4-NP to 4-AP even after 10 cycles.

## 4. Discussion

In this study, we present a new approach for the synthesis of Pt/SnO_2_ catalysts using microwave radiation. In contrast to previous methods using organic precursors, we used Pt(IV) and Sn(IV) as inorganic precursors (namely H_2_PtCl_6_ and SnCl_4_) that allow the controlled formation of platinum particles dispersed on the anisotropic SnO_2_ support. In both syntheses, annealing at 400 °C in the air was performed as the final synthesis step. The XRD patterns (Figure 1) show the platinum maxima in all Pt/SnO_2_ samples. The Williamson-Hall diagram shows anisotropy of SnO_2_ (Table 3 and Figure 2), with SnO_2_ crystallite size of about 10 nm in the c-axis direction (< *00l* >) and about 5 nm in the a-axis direction (< *h00* >). SEM results confirm the anisotropic nature of SnO_2_ and show anisotropic, urchin-like SnO_2_ particles (Figure 4 and Figure S7). SEM backscattering results show that the PtNPs in the Pt/SnO_2_ samples are quite homogeneously dispersed at the micrometer level (Figure 3). However, at higher magnification (Figure 5), it can be seen that the PtNPs are heterogeneously dispersed, with areas that are very rich in platinum and areas that contain almost no platinum (Figure 5 and Figure 7). The results of the BET nitrogen adsorption–desorption isotherms (Figure 8) show that the BET surface area decreases with platinum loading, consistent with the formation of large compact platinum nanoparticles that decrease the SnO_2_ surface area. The results of pore volume distributions show that all samples are mesoporous, and the pore diameter is about 4 nm. The presence of ammonia during the synthesis of the Pt/SnO_2_ powder samples and the subsequent heating at 400 °C act as a templating process, leading to the formation of mesoporous SnO_2_ samples. These pores provide a large surface area and an interconnected network of voids that can be beneficial for various applications such as catalysis, adsorption, and gas sensing. In addition, the good dispersion of relatively large platinum nanoparticles (PtNPs) may indicate a suitable surface area for catalytic reactions and potentially better accessibility for reactant molecules. Well-dispersed large PtNPs with high metal character could provide more active sites for reaction, contributing to the observed high catalytic efficiency.

The XPS results (Figure 9 and Table 4) show that the average oxidation state (AOS) of platinum is relatively low, with a minimum of 0.4 for sample SP3 and a maximum of 1.25 for sample SP15. On the other hand, the AOS of PtNPs determined in previous work, with a size of about 1 nm (see Ref [18,20] and Table 4), is about two to four times higher than the values determined in this work. It is well known that non-stoichiometry dominates with decreasing particle size. For example, Fe_3_O_4_ (stoichiometric magnetite) with a size of about 3 nm or less has a similar stoichiometry to γ-Fe_2_O_3_ (maghemite) [21,22,23]. TiO_2_ (titanium dioxide) nanoparticles with a size of about 4 nm can also deviate from the ideal stoichiometry [24,25]. PtNPs with a size of about 1 nm obtained in previous work (Table 4 and Refs. [18,20]) exhibit deviations from the ideal stoichiometry of the Pt metal. These small PtNPs are prone to oxidation [26,27,28,29], leading to a behavior that resembles that of sub-stoichiometric platinum oxide (PtO_x_) or other surface-bound species. In contrast, the large PtNPs obtained in this work (Figure 3 and Figure 6) have a stoichiometry much closer to the Pt metal (Table 4).

The ^119^Sn Mössbauer spectroscopy result shows that the ^119^Sn isomer shift values of the samples are all slightly above zero, indicating the presence of a small excess of electron density at the 5s level of Sn^4+^ compared to our SnO_2_ reference. This excess could be due to lattice defects, such as oxygen vacancies in the present samples. The decrease in isomer shifts in response to an increase in Pt concentration (Figure 11) can then be interpreted as a gradual decrease in electron density at the 5s level of Sn^4+^ due to the involvement of Pt in the formation of the electronic structure of SnO_2_. Since the electronegativity of Pt is higher than that of tin, it is plausible to expect that the incorporation of Pt into the bulk crystal structure or its attachment to the surface layer of SnO_2_ particles (e.g., as shown in [30]) exerts such an influence on the electronic structure of the host particles. Overall, the correlation between the ^119^Sn isomeric shift of SnO_2_ and the Pt concentration used suggests that (at least some of the) Pt atoms are in direct electronic contact with SnO_2_ particles.

The catalytic activity of the synthesized Pt/SnO_2_ samples was investigated using a model reaction of the catalytic reduction of 4-nitrophenol (4-NP) to 4-aminophenol (4-AP) [18,31,32,33]. The reduction of 4-NP to 4-AP is an oxidation-reduction (redox) reaction in which the nitro group (-NO_2_) of 4-NP is reduced to an amino group (-NH_2_) of 4-AP. The catalytic reduction of 4-NP to 4-AP is a well-studied model reaction, but the actual mechanism is still controversial. For instance, Wunder et al. [34] proposed a Langmuir-Hinshelwood mechanism in which borohydride ions facilitate the transfer of surface hydrogen species to nanoparticles, allowing reversible adsorption of 4-NP molecules, their reduction by adsorbed hydrogen, and subsequent desorption of 4-AP products, driving the catalytic cycle. Gu et al. [35] emphasized that 4-nitrophenol is first reduced to 4-nitrosophenol and then rapidly converted to 4-hydroxylaminophenol (Hx), which is the only stable intermediate. In the second step, Hx is reduced to the final product, 4-aminophenol. All reactions take place on the surface of the metal particles. Iben Ayad et al. [36] studied the reduction of 4-nitrophenol using pseudo-first-order kinetics and fitting to a Langmuir–Hinshelwood model and discovered two effective catalytic mechanisms involving surface-mediated hydrogen and electron transfer on metallic nanoparticles. The reaction involves sequential adsorption of reactants, formation of hydrogen radicals, and generation of 4-aminophenol via intermediate species, which ends with separation of the product and allows initiation of a new catalytic cycle. Zhao et al. [37] challenge the conventional understanding of 4-nitrophenol reduction by proposing a new mechanism that emphasizes the central role of protic solvents, particularly water, over NaBH_4_ as a hydrogen source. Their experiments with isotopically labeled solvents and reducing agents show that water is essential for the catalytic reduction of 4-aminophenol, with the hydrogen atoms originating from the solvent itself, challenging the established role of NaBH_4_. This proposed dynamic interaction between solvent, metal catalyst, and reactants shows that the reduction process relies on the protic solvent and opens a new perspective on this catalytic reaction.

The platinum-containing samples (samples SP1 to SP15) completely catalytically reduced 4-NP to 4-AP between 10 and 80 min (Figure 12). The higher catalytic activity of samples SP5, SP10, and SP15, which contained (NH_4_)_2_PtCl_6_, compared to samples SP1 and SP3, which did not contain (NH_4_)_2_PtCl_6_, indicates the positive effect of this impurity on the catalytic reduction of 4-NP to 4-AP. The sample containing 5 mol% Pt (sample SP5) showed the highest catalytic activity for the reduction of 4-NP to 4-AP with an apparent rate constant *k* = 0.59 × 10^–2^ s^–1^, so 5 mol% is an optimal platinum loading on the SnO_2_ support under the present experimental conditions. In general, the catalytic activity of Pt on metal oxide supports depends on several factors, such as platinum loading, dispersion of platinum, availability of platinum on the surface, and interaction of platinum with reducible metal oxide supports. Another very important property of the catalyst is its recyclability (reusability). The reusability test (Figure 13) showed that the best catalyst (sample SP5) is exceptionally robust and durable, with very high efficiency (81.4%) in reducing 4-NP to 4-AP after 10 cycles. The ability to reuse a catalyst multiple times not only offers economic advantages but is also consistent with the principles of sustainable and environmentally friendly chemical processes. Catalyst reusability is an essential criterion in catalyst development and selection because it directly affects the overall efficiency, cost, and environmental impact of chemical processes.

In the context of the catalytic reduction of 4-nitrophenol (4-NP) to 4-aminophenol (4-AP), several studies have reported remarkable catalysts and their respective results. For instance, Na et al. [38] presented a novel reusable catalyst consisting of platinum nanoparticles (PtNPs) on layered double hydroxide nanosheets (LDH). Their catalyst showed promising catalytic efficiency, achieving 92% conversion within 30 min. However, Na et al. performed reusability tests for only five cycles, making it difficult to determine the sustained reusability of the PtNP-LDH sample. Pandey et al. [39] synthesized PtNPs stabilized using guar gum and achieved over 90% conversion from 4-NP to 4-AP in 7 min. Although their catalyst exhibited high efficiency, the amount used was larger than that of our SP5 sample. It is noteworthy that no reusability tests were performed in their study. Ullah et al. [40] used phytochemicals for the biofabrication of PtNPs, which exhibited good catalytic efficiency. However, the conversion rate from 4-NP to 4-AP was less than 90%, with an apparent rate constant *k*_app_ = 0.2 × 10^–2^ s^–1^. Unfortunately, their study did not include information on the reusability of the catalyst. Bogireddy et al. [41] synthesized 2D platinum superstructures that exhibited efficient catalytic activity in the reduction of 4-NP to 4-AP as well as methylene blue and their mixture. Their self-assembled PtNPs showed promising results, although the evaluation of their reusability was limited to only five cycles. In contrast, in the present work, a conversion rate of over 96% was maintained after five cycles and 81.4% after 10 cycles. Ko et al. [42] investigated PtNP-[C_60_]fullerene nanowhisker composites for the reduction of 4-NP. While these composites showed catalytic activity at different temperatures, the catalyst exhibited higher performance at room temperature. Nevertheless, the apparent rate constants remained relatively low compared to our study. Liu et al. [43] described a method for embedding PtNPs in mesoporous carbon spheres that resulted in improved catalytic performance. Their Pt/C composites were found to be recyclable and achieved 80% conversion after six cycles. However, our SP5 sample surpassed their results by still achieving a conversion rate of over 80% after ten cycles. In summary, the catalytic reduction of 4-NP to 4-AP has been studied by several researchers, each presenting innovative catalysts with different efficiencies. Our current work with the SP5 catalyst showed exceptional results by achieving both high conversion rates and sustained efficiency over ten cycles. This exceeds the performance of catalysts presented in previous studies and underscores the potential impact of our approach.

## 5. Conclusions

In this study, a new approach for the synthesis of Pt/SnO_2_ catalysts using microwave irradiation is presented. The use of inorganic precursors (H_2_PtCl_6_ and SnCl_4_) enables the controlled formation of platinum particles on the anisotropic SnO_2_ support. The PtNPs are homogeneously dispersed in the micrometer range. The Pt/SnO_2_ samples exhibit mesoporous properties that are advantageous for applications in catalysis, adsorption, and gas sensing.

XPS analysis shows that the oxidation state of Pt is relatively low, and the stoichiometry of the large PtNPs obtained in this study is close to that of the Pt metal. The ^119^Sn-Mössbauer spectroscopy indicates electronic interactions between Pt and SnO_2_ particles.

The Pt/SnO_2_ catalysts show excellent catalytic activity in the reduction of 4-nitrophenol to 4-aminophenol, with a 5-mol% Pt loading showing optimal performance.

The unique anisotropic structure of the SnO_2_ support, combined with the controlled dispersion of PtNPs, resulted in a robust and durable Pt/SnO_2_ catalyst with very high efficiency in reducing 4-NP to 4-AP even after 10 cycles. These findings open new avenues for the design and fabrication of advanced catalytic systems for future catalytic and environmental applications.

## Figures and Tables

**Table 1:** The annotation of samples and corresponding volumes of 2.0 mol dm^−3^ (2 M) SnCl_4_ and 2.0 mol dm^−3^ (2 M) H_2_PtCl_6_ stock solutions were added to the volumetric flask and filled with water up to the 20 mL mark. The [Pt^IV^/(Pt^IV^ + Sn^IV^)] represents the molar fractions of initially added platinum and tin ions in precursor suspensions.

Sample	*V*(SnCl_4_ Stock Solution)/mL	*V*(H_2_PtCl_6_ Stock Solution)/µL	[Pt^IV^/(Pt^IV^ + Sn^IV^)]
SP0	3.00	0	0.00
SP1	2.97	30	0.01
SP3	2.91	90	0.03
SP5	2.85	150	0.05
SP10	2.70	300	0.10
SP15	2.55	450	0.15

**Table 2:** The results of phase analysis of the samples SP0, SP1, SP3, SP5, SP10, and SP15.

Sample	*x* (Pt)	Phase Composition
SP0	0	SnO_2_
SP1	0.01	SnO_2_ + Pt
SP3	0.03	SnO_2_ + Pt
SP5	0.05	SnO_2_ + Pt + (NH_4_)_2_PtCl_6_
SP10	0.10	SnO_2_ + Pt + (NH_4_)_2_PtCl_6_
SP15	0.15	SnO_2_ + Pt + (NH_4_)_2_PtCl_6_

Description: SnO_2_ = phase structurally closely related to cassiterite (ICDD Card no. 41-1445); Pt = phase structurally closely related to cubic platinum (ICDD Card no. 04-802); (NH_4_)_2_PtCl_6_ = phase structurally closely related to cubic ammonium platinum chloride (ICDD Card no. 7-218).

**Table 3:** The *hkl* indices, the 2*θ* positions, the FWHM values, and the *D_hkl_* values (estimated from Scherrer equation) of the most prominent well-separated diffraction lines of SnO_2_ phase in samples SP0, SP1, SP3, SP5, SP10, and SP15.

Sample	Phase	*h k l*	2*θ*/°	FWHM/°	*D_hkl_*/nm
SP0	SnO_2_	1 1 0	26.66	1.49	5.5(2)
		1 0 1	33.90	1.06	7.8(3)
		2 0 0	38.18	1.79	4.7(1)
		2 1 1	51.81	1.62	5.5(2)
		2 2 0	54.91	1.64	5.5(2)
		0 0 2	57.87	0.83	11.0(5)
		2 0 2	71.27	1.40	7.0(3)
SP1	SnO_2_	1 1 0	26.65	1.69	4.8(1)
		1 0 1	33.91	1.21	6.9(2)
		2 0 0	38.21	1.91	4.4(1)
		2 1 1	51.83	1.59	5.6(2)
		2 2 0	54.88	1.71	5.2(2)
		0 0 2	57.85	0.89	10.2(4)
		2 0 2	71.30	1.23	8.0(3)
SP3	SnO_2_	1 1 0	26.65	1.61	5.1(2)
		1 0 1	33.90	1.13	7.3(3)
		2 0 0	38.23	1.72	4.9(1)
		2 1 1	51.83	1.67	5.3(2)
		2 2 0	54.91	1.58	5.7(2)
		0 0 2	57.86	0.83	11.0(5)
		2 0 2	71.30	1.44	6.8(3)
SP5	SnO_2_	1 1 0	26.64	1.76	4.6(1)
		1 0 1	33.86	1.26	6.6(2)
		2 0 0	38.16	1.77	4.7(1)
		2 1 1	51.81	1.76	5.0(2)
		2 2 0	54.88	1.71	5.2(2)
		0 0 2	57.82	0.89	10.2(5)
		2 0 2	71.23	1.34	7.3(3)
SP10	SnO_2_	1 1 0	26.66	1.42	5.8(2)
		1 0 1	33.91	0.92	9.0(3)
		2 0 0	38.18	1.71	4.9(1)
		2 1 1	51.83	1.43	6.1(2)
		2 2 0	54.83	1.50	6.0(2)
		0 0 2	57.90	0.66	13.7(5)
		2 0 2	71.32	1.06	9.2(3)
SP15	SnO_2_	1 1 0	26.66	1.29	6.3(2)
		1 0 1	33.90	0.81	10.2(4)
		2 0 0	38.21	1.65	5.1(1)
		2 1 1	51.81	1.24	7.1(3)
		2 2 0	54.77	1.35	6.6(2)
		0 0 2	57.83	0.75	12.1(5)
		2 0 2	71.34	0.93	10.5(4)

**Table 4:** Average oxidation state (AOS) of platinum (Pt) and tin (Sn) calculated on the basis of XPS results ^i^.

Sample	AOS of Pt (This Work)	AOS of Pt ^ii^ (Ref. [18])	AOS of Sn (This Work)	AOS of Sn ^ii^ (Ref. [18])
SP1	0.74	1.69	3.93	3.49
SP3	0.40	1.69	3.94	3.22
SP5	0.80	1.73	3.92	3.56
SP10	0.85	2.11	3.94	3.50
SP15	1.25	-	3.84	-

^i^ The average XPS oxidation state of Pt is calculated using the following equation: Pt (average oxidation state) = mole fraction Pt(IV) × 4 + mole fraction Pt(II) × 2 + mole fraction Pt(0) × 0, where mole fraction equals XPS proportion %/100 and these values are given above in Table S1. The same model is used for the average oxidation state of Sn. ^ii^ The average XPS oxidation states of Pt and Sn were obtained in a previous work [18].

**Table 5:** Room temperature ^119^Sn Mössbauer parameters obtained via the fitting of the spectra displayed in Figure 10 to a symmetrical quadrupole doublet of Lorentzians. *δ* and Δ denote the ^119^Sn isomer shift and quadrupole splitting, respectively, whereas *W* stands for the FWHM line width of the individual Lorentzians of the doublet. Numbers between parentheses denote the standard fit error (1σ) in the last digit(s). See also Figure 11 regarding the isomer shift values.

Pt mol%	*δ*/mm s^–1^	Δ/mm s^–1^	*W*/mm s^–1^
0	0.0121(10)	0.541(3)	1.028(5)
1	0.0121(11)	0.520(4)	1.008(5)
3	0.0134(12)	0.521(4)	1.012(6)
5	0.0116(13)	0.518(5)	1.043(6)
10	0.0093(12)	0.519(4)	1.035(6)
15	0.0070(7)	0.516(2)	1.005(3)

## Data Availability

Not applicable.

## Supplementary Materials

The following supporting information can be downloaded at: https://www.mdpi.com/article/10.3390/nano13172481/s1, Figure S1: UV-Vis spectra of the aqueous solution of pure 4-nitrophenol and 4-nitrophenolate ions after the addition of NaBH_4_.; Figure S2: Catalytic reduction of 4-nitrophenol (4-NP) to 4-aminophenol (4-AP) as a function of time using samples SP0 containing no platinum.; Table S1: The peak positions and relative proportion (%) of Pt^4+^, Pt^2+^, Pt^0^, Sn^4+^ and Sn^2+^ in the synthesized samples based on the deconvoluted Pt 4f and Sn 3d spectra.; Figures S3 to S6: SEM EDS results of samples Sp3, SP5, SP10 and SP15; Figure S7: SEM EDS results of urchin-like particle in sample SP5.
