# Supplementary material for: Microwave-Assisted Synthesis of Pt/SnO2 for the Catalytic Reduction of 4-Nitrophenol to 4-Aminophenol
Source: Nanomaterials (Basel). 2023 Sep 2;13(17):2481. doi: 10.3390/nano13172481 (PMC10489642; doi:10.3390/nano13172481)
Supplement: Supplementary file 1 [file nanomaterials-13-02481-s001.zip › nanomaterials-2577613-supplementary.pdf]

Supplementary materials

Microwave-Assisted Synthesis of Pt/SnO_2 for the Catalytic Reduction of 4-Nitrophenol to 4-Aminophenol

Izabela Đurasović [1], Goran Štefanić [1], Goran Dražić [2], Robert Peter [3], Zoltán Klencsár [4], Marijan Marciuš [5], Tanja Jurkin [6], Mile Ivanda [1], Sándor Stichleutner [4], Marijan Gotić [1,*]

[1] Laboratory for Molecular Physics and Synthesis of New Materials, Division of Materials Physics, Ruđer Bošković Institute, Bijenička c. 54, 10000 Zagreb, Croatia; idjuras@irb.hr (I.Đ.); goran.stefanic@irb.hr (G.Š.); ivanda@irb.hr (M.I.); gotic@irb.hr (M.G.)
[2] National Institute of Chemistry, Hajdrihova 19, SI-1001 Ljubljana, Slovenia; goran.drazic@ki.si
[3] Department of Physics, University of Rijeka, Radmile Matejčić 2, 51000 Rijeka, Croatia; rpeter@phy.uniri.hr
[4] Nuclear Analysis and Radiography Department, Centre for Energy Research, 1121 Budapest, Hungary; klencsar.zoltan@ek-cer.hu (Z.K.); stichleutner@izotop.hu (S.S.)
[5] Division of Materials Chemistry, Ruđer Bošković Institute, Bijenička c. 54, 10000 Zagreb, Croatia; marijan.marcius@irb.hr (M.M.)
[6] Radiation Chemistry and Dosimetry Laboratory, Division of Materials Chemistry, Ruđer Bošković Institute, Bijenička c. 54, 10000 Zagreb, Croatia; tjurkin@irb.hr (T.J.)

***Correspondence:** gotic@irb.hr (M.G.)

Table of contents:

Fig. S1 UV-Vis spectra of the aqueous solution of pure 4-nitrophenol and 4-nitrophenolate ions after addition of $NaBH_4$.

Fig. S2 Catalytic reduction of 4-nitrophenol (4-NP) to 4-aminophenol (4-AP) as a function of time using samples SP0 containing no platinum.

Table S1 The peak positions and relative proportion (%) of Pt^{4+}, Pt^{2+}, Pt^{0}, Sn^{4+} and Sn^{2+} in the synthesized samples based on the deconvoluted Pt 4f and Sn 3d spectra.

Figs. S3 to S6 SEM EDS results of samples Sp3, SP5, SP10 and SP15.

Fig. S7 SEM EDS results of urchin-like particle in sample SP5.

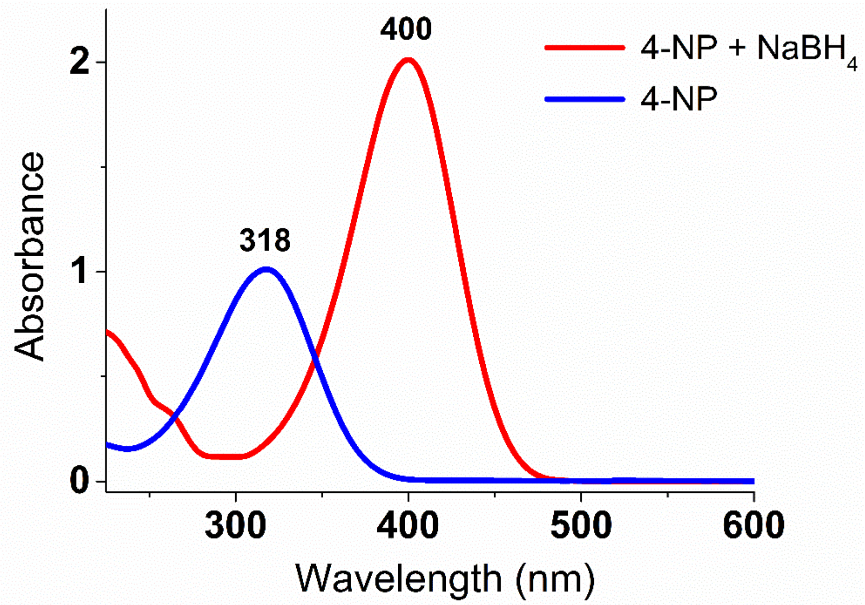

Fig. S1 UV-Vis spectra of the aqueous solution of pure 4-nitrophenol with the maximum at 318 nm and 4-nitrophenolate ions after addition of $NaBH_4$ (alkaline aqueous solution) with maximum at 400 nm.

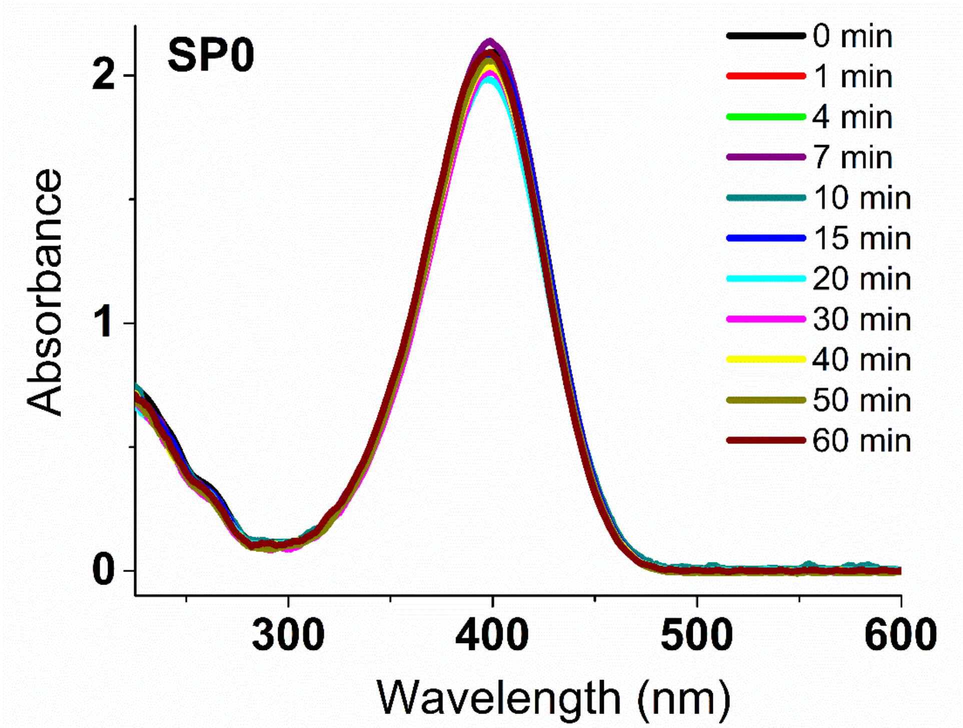

Fig. S2 Catalytic reduction of 4-nitrophenol (4-NP) to 4-aminophenol (4-AP) as a function of time using sample SP0, which does not contain platinum. This sample is completely inactive for the reduction of 4-NP to 4-AP.

Table S1 The peak positions and relative proportions (%) of Pt^{4+}, Pt^{2+}, Pt^{0}, Sn^{4+} and Sn^{2+} in the synthesized samples based on the deconvoluted Pt 4f and Sn 3d spectra.

Sample	Electron configuration	Binding energy / eV	Pt / %	Electron configuration	Binding energy / eV	Sn / %
**SP1**	Pt^{4+} $4f_{5/2}$	–	Pt^{4+} / 0	Sn^{4+} $3d_{3/2}$	495.2	Sn^{4+} / 96.6
	Pt^{4+} $4f_{7/2}$	–		Sn^{4+} $3d_{5/2}$	486.8	
	Pt^{2+} $4f_{5/2}$	75.7	Pt^{2+} / 36.8	Sn^{2+} $3d_{3/2}$	493.8	Sn^{2+} / 3.4
	Pt^{2+} $4f_{7/2}$	72.5		Sn^{2+} $3d_{5/2}$	485.4	
	Pt^{0} $4f_{5/2}$	74.0	Pt^{0} / 63.2	Sn^{0} $3d_{3/2}$	–	Sn^{0} / 0
	Pt^{0} $4f_{7/2}$	70.8		Sn^{0} $3d_{5/2}$	–	
	**Pt 4f**	**eV**	**Pt / %**	**Sn 3d**	**eV**	**Sn / %**
**SP3**	Pt^{4+} $4f_{5/2}$	–	Pt^{4+} / 0	Sn^{4+} $3d_{3/2}$	495.1	Sn^{4+} / 97.1
	Pt^{4+} $4f_{7/2}$	–		Sn^{4+} $3d_{5/2}$	486.6	
	Pt^{2+} $4f_{5/2}$	75.7	Pt^{2+} / 19.9	Sn^{2+} $3d_{3/2}$	493.6	Sn^{2+} / 2.9
	Pt^{2+} $4f_{7/2}$	72.4		Sn^{2+} $3d_{5/2}$	485.2	
	Pt^{0} $4f_{5/2}$	74.0	Pt^{0} / 80.1	Sn^{0} $3d_{3/2}$	–	Sn^{0} / 0
	Pt^{0} $4f_{7/2}$	70.8		Sn^{0} $3d_{5/2}$	–	
	**Pt 4f**	**eV**	**Pt / %**	**Sn 3d**	**eV**	**Sn / %**
**SP5**	Pt^{4+} $4f_{5/2}$	77.5	Pt^{4+} / 7.8	Sn^{4+} $3d_{3/2}$	495.2	Sn^{4+} / 96.1
	Pt^{4+} $4f_{7/2}$	74.3		Sn^{4+} $3d_{5/2}$	486.8	
	Pt^{2+} $4f_{5/2}$	75.8	Pt^{2+} / 24.6	Sn^{2+} $3d_{3/2}$	493.6	Sn^{2+} / 3.9
	Pt^{2+} $4f_{7/2}$	72.5		Sn^{2+} $3d_{5/2}$	485.2	
	Pt^{0} $4f_{5/2}$	74.1	Pt^{0} / 67.6	Sn^{0} $3d_{3/2}$	–	Sn^{0} / 0
	Pt^{0} $4f_{7/2}$	70.8		Sn^{0} $3d_{5/2}$	–	
	**Pt 4f**	**eV**	**Pt / %**	**Sn 3d**	**eV**	**Sn / %**
**SP10**	Pt^{4+} $4f_{5/2}$	77.5	Pt^{4+} / 8.3	Sn^{4+} $3d_{3/2}$	495.1	Sn^{4+} / 97.1
	Pt^{4+} $4f_{7/2}$	74.3		Sn^{4+} $3d_{5/2}$	486.7	
	Pt^{2+} $4f_{5/2}$	75.8	Pt^{2+} / 25.8	Sn^{2+} $3d_{3/2}$	493.7	Sn^{2+} / 2.9
	Pt^{2+} $4f_{7/2}$	72.6		Sn^{2+} $3d_{5/2}$	485.3	
	Pt^{0} $4f_{5/2}$	74.2	Pt^{0} / 65.9	Sn^{0} $3d_{3/2}$	–	Sn^{0} / 0
	Pt^{0} $4f_{7/2}$	70.9		Sn^{0} $3d_{5/2}$	–	
	**Pt 4f**	**eV**	**Pt / %**	**Sn 3d**	**eV**	**Sn / %**
**SP15**	Pt^{4+} $4f_{5/2}$	77.5	Pt^{4+} / 16.3	Sn^{4+} $3d_{3/2}$	495.3	Sn^{4+} / 92.2
	Pt^{4+} $4f_{7/2}$	74.3		Sn^{4+} $3d_{5/2}$	486.8	
	Pt^{2+} $4f_{5/2}$	75.7	Pt^{2+} / 29.8	Sn^{2+} $3d_{3/2}$	493.4	Sn^{2+} / 7.8
	Pt^{2+} $4f_{7/2}$	72.5		Sn^{2+} $3d_{5/2}$	485.0	
	Pt^{0} $4f_{5/2}$	74.1	Pt^{0} / 53.9	Sn^{0} $3d_{3/2}$	–	Sn^{0} / 0
	Pt^{0} $4f_{7/2}$	70.8		Sn^{0} $3d_{5/2}$	–	

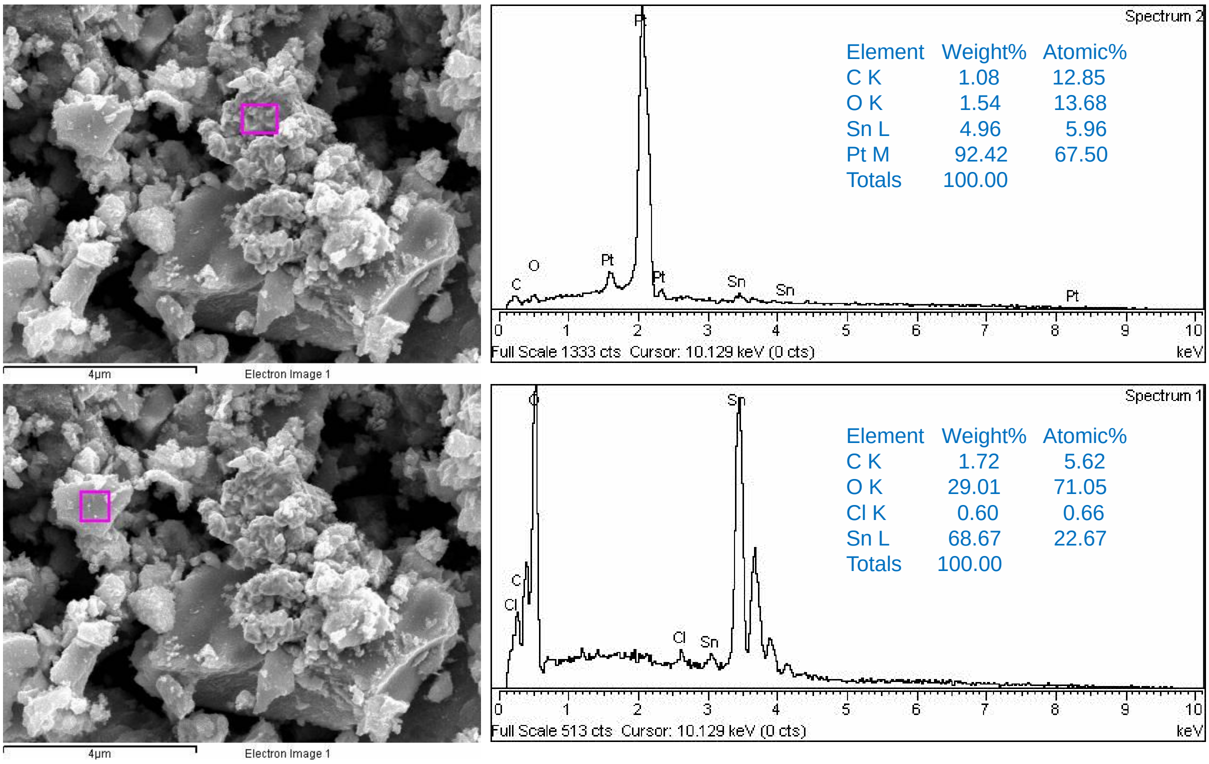

Fig. S3 SEM EDS results of sample SP3. EDS analyses were taken from two different marked sites showing the platinum rich region (upper panel) and SnO_2 rich region (lower panel).

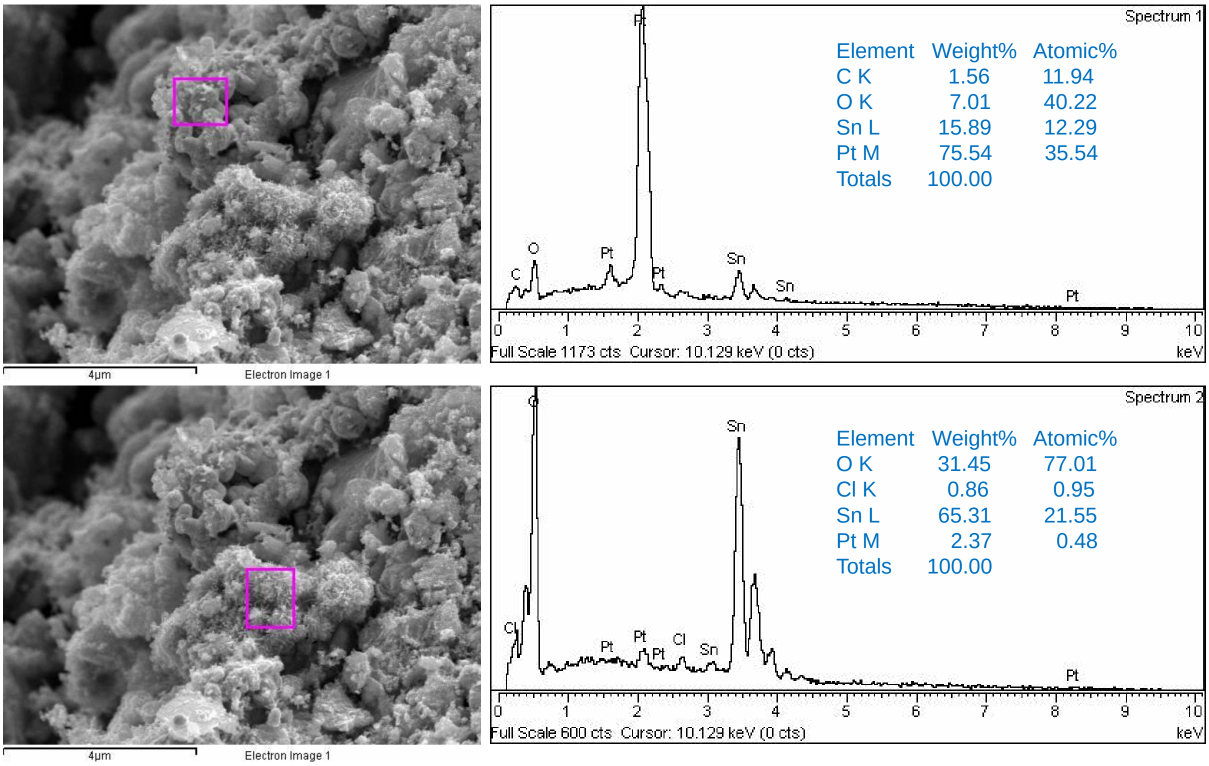

Fig. S4 SEM EDS results of sample SP5. EDS analyses were taken from two different marked sites showing the platinum rich region (upper panel) and SnO_2 rich region (lower panel).

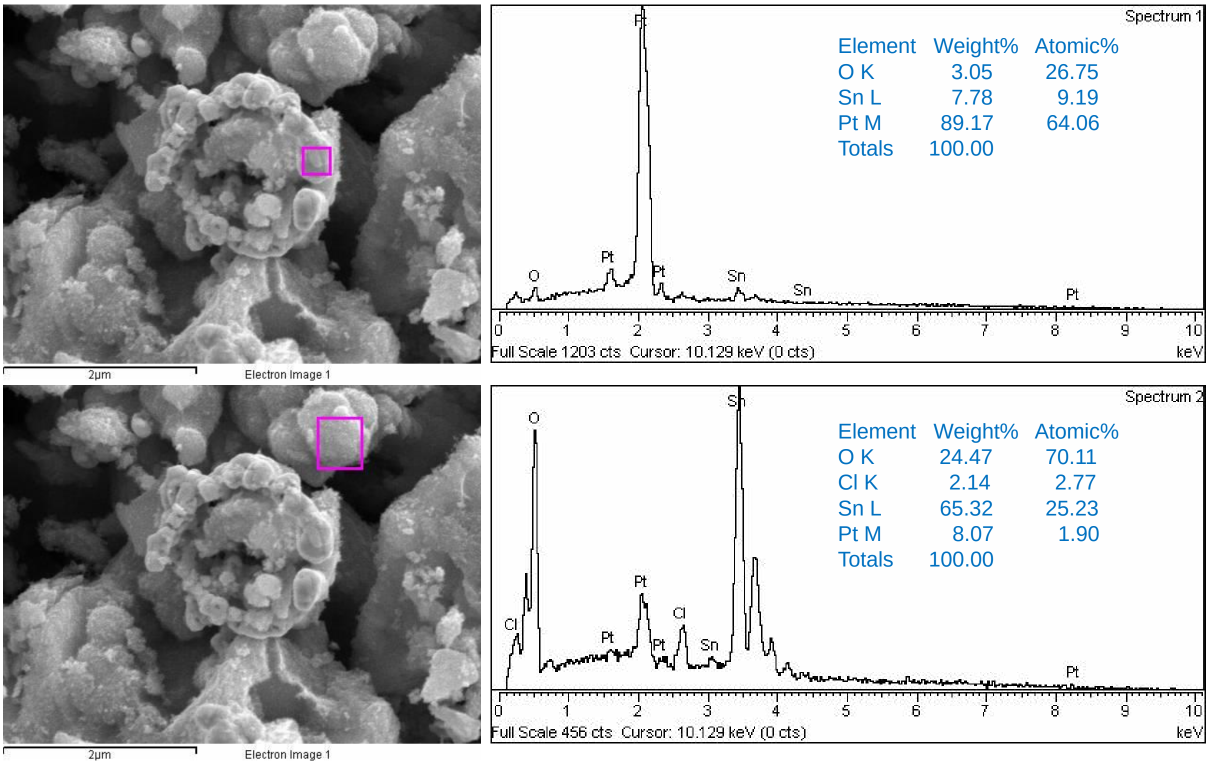

Fig. S5 SEM EDS results of sample SP10. EDS analyses were taken from two different marked sites showing the platinum rich region (upper panel) and SnO_2 rich region (lower panel).

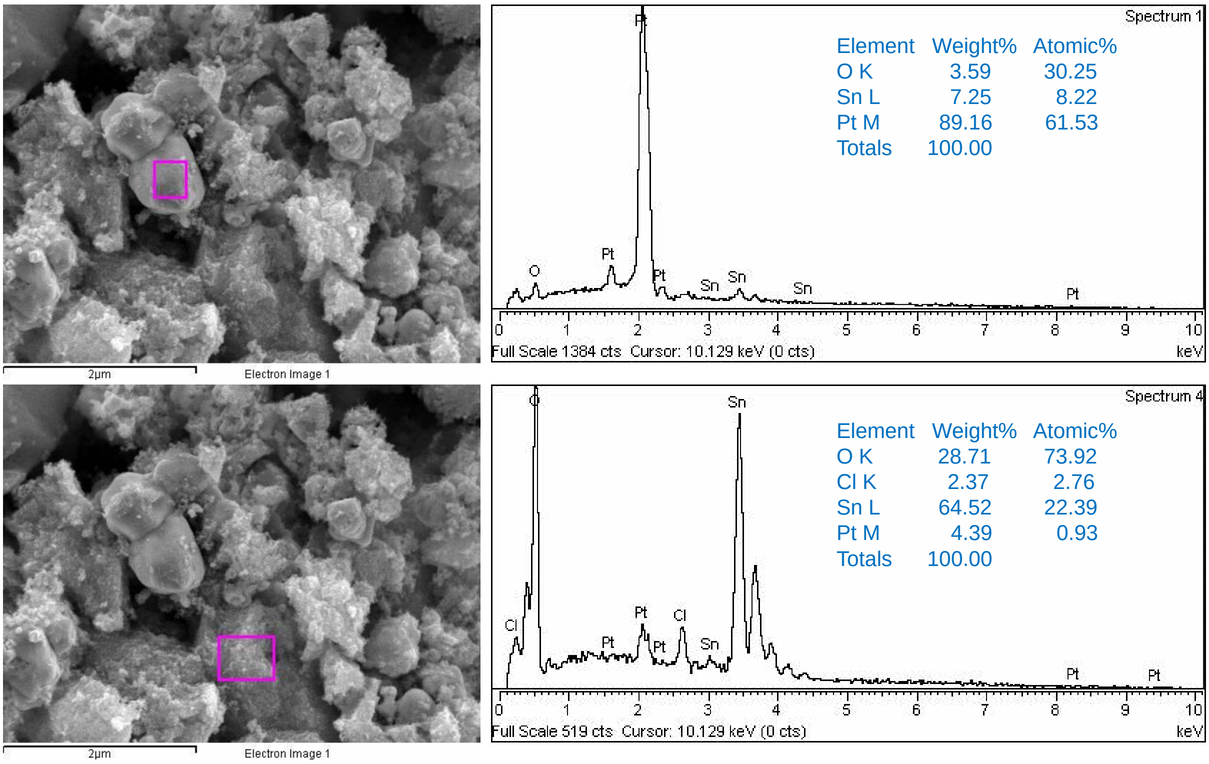

Fig. S6 SEM EDS results of sample SP15. EDS analyses were taken from two different marked sites showing the platinum rich region (upper panel) and SnO_2 rich region (lower panel).

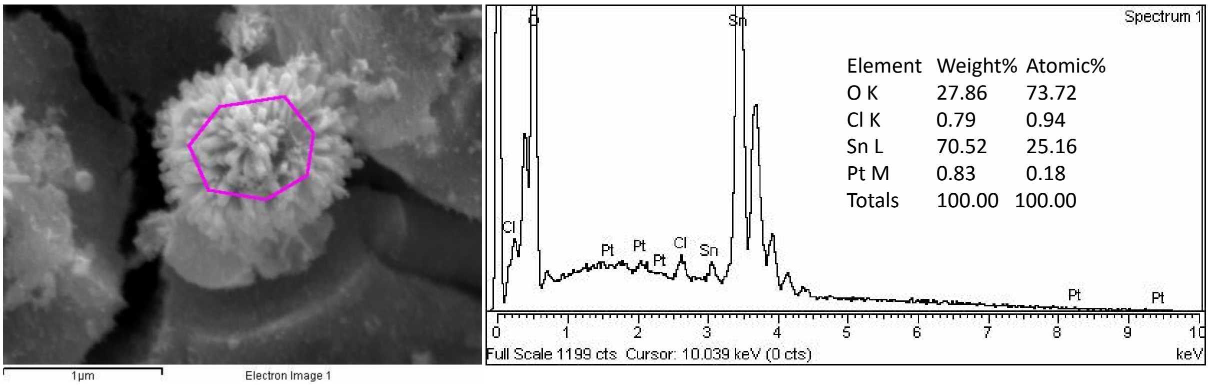

Fig. S7 SEM EDS results of urchin-like particle in sample SP5. The image shows anisotropic urchin-like particles. EDS analyses taken from the marked sites showing that the urchin-like particle mainly consisted of SnO_2.
